# Early postnatal alterations in follicular stress response and survival in a mouse model of Classic Galactosemia

**DOI:** 10.1186/s13048-022-01049-2

**Published:** 2022-11-21

**Authors:** Synneva Hagen-Lillevik, Joshua Johnson, Kent Lai

**Affiliations:** 1 Department of Pediatrics, University of Utah School of Medicine, 295 Chipeta Way, Salt Lake City, UT 84108 USA; 2 Department of Nutrition and Integrative Physiology, University of Utah College of Health, 250 South 1850 East Room 214, Salt Lake City, UT 84112 USA; 3 Division of Reproductive Sciences, Division of Reproductive Endocrinology and Infertility, Department of Obstetrics and Gynecology, University of Colorado Denver (AMC), Building RC2, Room P15 3103, Mail Stop 8613, Aurora, CO 80045 USA

**Keywords:** Classic Galactosemia, Primary ovarian insufficiency, Eukaryotic initiation factor 2 alpha (eIF2ɑ), Integrated stress, Ovary, Oocyte, Translational control, Aging, Follicle, Granulosa cells

## Abstract

Primary ovarian insufficiency is characterized by accelerated loss of primordial follicles, which results in ovarian failure and concomitant menopause before age 40. About 1–3% of females in the general population are diagnosed with POI; however, greater than 80% of females with the inherited disease Classic Galactosemia will develop POI. Classic Galactosemia is caused by mutations in the *GALT* gene encoding the enzyme galactose-1 phosphate uridylyltransferase. While dietary restriction of galactose is lifesaving in the neonatal period, the development of complications including primary ovarian insufficiency is not mitigated. Additionally, the pattern(s) of follicle loss have not been completely characterized. The chronic accumulation of aberrant metabolites such as galactose-1-phosphate and galactitol are suspected culprits in the development of the sequelae, yet the mechanisms remain elusive.

Our group uses a *GalT* gene-trapped mouse model to study the pathophysiology of primary ovarian insufficiency in Classic Galactosemia. We recently showed that differences in the Integrated Stress Response pathway occur in mutant ovaries that likely contribute to their primary ovarian insufficiency phenotype. Using immunofluorescent staining of histological sections of ovaries at progressive ages, we saw evidence of altered Integrated Stress Response activity in granulosa cells and primordial oocytes consistent with accelerated primordial follicle growth activation, aberrant DNA damage and/or repair, and increased cellular stress/death. Overall, our findings indicate that abnormal Integrated Stress Response in the Classic Galactosemia model ovary results in accelerated primordial follicle growth activation, sometimes referred to as “burnout.” These aberrant early events help further clarify when/how the primary ovarian insufficiency phenotype arises under galactosemic conditions.

## Introduction

Classic Galactosemia (CG) is an inherited disease caused by mutations in the *GALT* gene encoding the enzyme galactose-1 phosphate uridylyltransferase in the galactose metabolic pathway [1]. Dietary restriction of galactose is lifesaving in the neonatal period. However, the development of severe complications such as cognitive impairments, tremor, ataxia, speech/language delays, growth restriction, and for 80–90% of females, primary ovarian insufficiency (POI), is not mitigated by this restriction [2, 3]. Given the enzymatic function of GALT, the chronic accumulation, and resulting cellular toxicity of aberrant metabolites such as galactose-1 phosphate (gal-1P) and galactitol in its absence are the suspected culprits in the development of the sequelae. Despite elevations in these metabolites, specific mechanisms of the pathology in CG remain elusive [4–6]. Data from human patients and several models of GALT deficiency including mice, fruit flies, and zebrafish show that the presence of such toxic metabolites correlates with upregulation of the Unfolded Protein Response (UPR), oxidative stress, altered glycosylation, and changes in global gene expression [7–12].

As mentioned, POI affects most females with CG [3]. POI is defined as the loss of ovarian function with concomitant menopause prior to age 40 [13]. The early onset of POI in CG means that it is particularly severe for those affected; it is characterized by a greatly accelerated loss of the ovarian follicle reserve, and correspondingly, abnormalities in hormone levels (low anti-Mullerian hormone [AMH], high follicle stimulating hormone [FSH], low estradiol) as early as childhood, delayed or absent puberty, and oligo/amenorrhea in adolescence [14]. Hormonal replacement therapy (HRT) can be used to address the hypo-estrogenic consequences of POI, such as cardiovascular compromise, adrenal insufficiency, increased adiposity, and decreased bone mineral density [15, 16]. However, addressing these consequences with HRT does not alleviate the early demise of the ovarian function and resulting infertility. The added psychological stress of fertility loss and the overall costs associated with long-term management of menopause add greatly to the impact of CG for affected females [16, 17].

Human females are born with dormant primordial follicles consisting of immature eggs (oocytes) surrounded by somatic cells (granulosa cells) that number in the hundreds of thousands [18]. The remaining number of primordial follicles at any time is considered the ovarian reserve. The depletion from this reserve occurs due to folliculogenesis, which is the growth and development of dormant primordial follicles to a terminal stage where they can ovulate a mature egg. The process consists of a regular transition of immature primordial follicles to successive primary, secondary, and antral preovulatory growth phases. Most follicles will not reach ovulation but will instead perish through a process termed atresia. Thus, quiescence of primordial follicles and the rates of follicle loss to atresia are integral in determining the duration of ovarian function during a female’s lifespan. Several cellular signaling pathways that respond to insults like oxidative damage, protein folding, and double stranded DNA breaks are known to be involved in folliculogenesis; disruptions in these signaling pathways can lead to accelerated primordial follicle depletion and POI [19–21].

As previously mentioned, the pattern(s) of follicle loss due to severe POI associated with CG have not been completely characterized. In human females with CG, there is evidence of normal appearing ovaries/follicles at birth and in early childhood. However, by adolescence, evidence of rapid primordial follicle depletion and lower numbers of developing follicles support postnatal insult [22, 23]. Nonetheless, the precise timing and mechanisms of immature follicle loss remain unknown. One feature of the aberrant cellular metabolism in CG is the production of deleterious cell metabolites that may compromise cell survival. One proposed mechanism of follicle loss is the phenomenon referred to as “burnout” in response to chemotherapeutic agents [24]. In follicle burnout, insult of the primordial reserve of follicles results in an instigation of accelerated growth activation (as evidenced by an initial increase in numbers of growing follicles), which is then followed by high levels of follicle atresia. Our analysis here included the possibility that the CG condition might induce a “burnout-like” outcome.

**Fig. 1:**
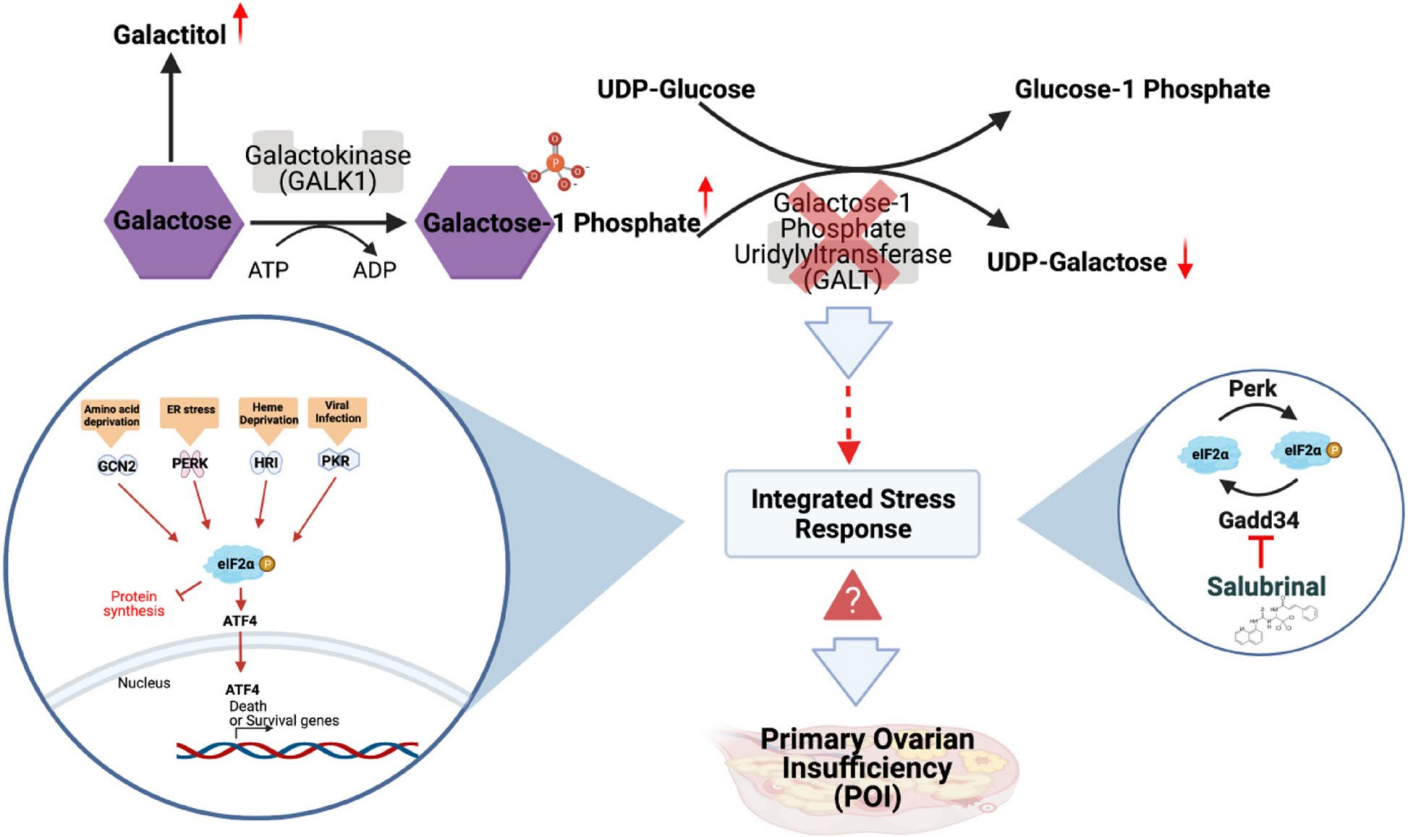
Leloir pathway of galactose metabolism. GALT deficiency leads to increased galactose-1 phosphate and decreased UDP-galactose. The Integrated Stress Response (ISR) is involved in the pathogenesis of primary ovarian insufficiency. Manipulating the phosphorylated state of eIF2ɑ with Salubrinal increases primordial follicle numbers and improves fertility and fecundity in a *GalT*KO mouse model of Classic Galactosemia

Our group uses a *GalT* gene-trapped (*GalT*KO) mouse model to study the pathophysiology of GALT deficiency in a physiologically relevant mammal [7, 25–29]. These animals phenocopy many features of CG seen in humans, including greatly accelerated ovarian aging (mouse “POI”). In recent work, we showed that alterations in the Integrated Stress Response (ISR) pathway occur in the ovaries of these mice in a way that likely contributes to the POI phenotype [30]. The ISR is characterized by the phosphorylation of eukaryotic transcription initiation factor alpha (eIF2ɑ) at Serine 51 (P-eIF2ɑ) by four kinases that are activated during specific cellular stress events such as DNA damage, heme deficiency, viral infection, low amino acids, and endoplasmic reticulum stress [31] (Fig. 1). Under low stress conditions, unphosphorylated eIF2ɑ initiates 5′ cap dependent mRNA translation and general protein synthesis in the cell [32, 33]. In contrast, the inactivating phosphorylation of eIF2ɑ when cells are under stress inhibits general protein translation and activates the translation of transcripts for proteins involved in the stress response and cellular repair mechanisms [34]. The phosphorylation of eIF2ɑ in the oocyte [35, 36] and granulosa cells [30] are thought to keep follicles quiescent until activation. P-eIF2ɑ levels were lower in *GalT*KO whole ovary specimens [30] suggesting that the ISR’s role in preserving primordial follicle quiescence is compromised under galactosemic conditions. This was assessed only in the sexually mature ovary, leaving open the question of whether the ISR pathway is dysregulated such that it impacts follicle development in early postnatal life [30].

The goal of this study was to determine the earliest timing of onset of altered ISR signaling in oocytes and granulosa cells of immature follicles in the context of galactosemia, and to expand our knowledge of the specific ovarian cell types involved. Here we report our findings of the early onset of molecular changes in primordial oocytes and granulosa cells of various stages of follicle development in juvenile mice.

## Methods

### *GalT* gene-trapped mouse model

All animal studies were conducted in full compliance by the guidelines outlined for the care and use of laboratory animals and were approved by the University of Utah Institutional Animal Care and Use Committee (IACUC). *GalT*KO mice used in this study were established as previously described; all animals were maintained on normal chow, and the genetic background of all mice was confirmed by genotyping using previously published protocols [25]. Table 1 summarizes the number of distinct ovary replicates from unique animals, as well as the number of cells or follicles evaluated in each experiment.

**Table 1:** For each genotype, the number of individual mice used to collect specimens is listed as *n* = the number of mice used. Numbers of replicates of follicle granulosa cell areas or oocytes that were measured in individual ovaries (from the indicated number of mice) are provided per sample where needed within parentheses

The Number of Ovary Replicates and the Number of Cells or Follicles Evaluated						
Fig. 2	**A**	**B**	**C**	**D, E**	**F**	**H, I**
WT	*n* = 5 (4,5,6,2,4)	*n *= 5 (3,6,5,2,4)	*n *= 2 (10,10)	*n* = 5 (4,6,2,2,6)	*n *= 5 (5,1,5,6,5)	*n* = 4 (4,2,4,1)
*GalT*KO	*n* = 5 (3,5,5,2,4)	*n *= 5 (5,6,7,3,3)	*n* = 3 (10,7,10)	*n *= 4 (3,1,1,3)	*n* = 3 (6,7,7)	*n* = 5 (9,2,4,9,1)
Fig. 3	**A**	**B**	**C**	**F**		
WT	*n *= 5 (13,16,14,2,5)	*n *= 5 (13,16,14,2,5)	*n* = 5	*n *= 5		
*GalT*KO	*n* = 5 (5,6,6,9,5,13)	*n *= 5 (5,6,6,9,5,13)	*n *= 3	*n *= 3		
Fig. 4	**A**	**B**	**C**	**F**	**H**	
WT	*n* = 5 (10,10,10,10,10)	*n *= 5 (10,10,10,10,10)	*n* = 2 (7,6)	*n* = 5 (10,10,9,10,10)	*n *= 5 (10,10,10,10,10)	
*GalT*KO	*n* = 5 (10,6,10,10,10)	*n *= 5 (10,6,10,10,10)	*n *= 2 (6,8)	*n *= 4 (10,10,10,10,10)	*n* = 3 (10,10,10)	
Fig. 5	**A**	**B**	**C**	**D**	**E**	
WT	*n *= 5 (24,17,12,24,14)	*n* = 5 (24,17,12,24,14)	*n* = 5 (23,17,17,16,21)	*n* = 5 (23,17,17,16,21)	*n* = 3 (10,10,10)	
*GalT*KO	*n* = 5 (20,18,9,12,14)	*n *= 5 (20,18,9,12,14)	*n *= 5 (22,22,11,38,25)	*n *= 5 (22,22,11,38,25)	*n* = 5 (10,10,11,10,10)	
Fig. 6	**A**	**B**	**C**	**D**		
WT	*n *= 5	*n *= 6	*n* = 6	*n *= 5		
*GalT*KO	*n* = 5	*n* = 6	*n *= 6	*n* = 5		

### Immunofluorescent studies

Ovaries were extracted from 5D, 14D, and 30D wildtype (WT) and *GalT*KO mice as described previously [29] and placed in 10% non-buffered formalin for 12 hours. The tissues were re-hydrated with 70% alcohol, embedded in paraffin, and sectioned by the Research Histology Core Facility of the ARUP Laboratories (Salt Lake City, UT, USA). Two cross sections of ovary five microns thick were placed on each slide, one for a secondary antibody only control. Cross sections were used to localize the expression of P-eIF2ɑ (Serine 51) (1:100; Cell Signaling Technologies, cat# 3398), binding immunoglobulin protein (BiP) (1:100; Cell Signaling Technologies, cat# 3177S), Cleaved Caspase-3 (Asp175) (CCASP3) (1:500; Cell Signaling Technologies, cat# 9664P), phospho-histone H2A.X (ser139) (ɣ-H2AX) (1:480; Cell Signaling Technologies, cat# 9718S), and proliferation marker protein Ki67 (Ki67) (1:200; Cell Signaling Technologies, cat# 12202S) in the ovary.

Briefly, after deparaffinization and rehydration, antigen retrieval was performed by incubating sections in either 10 mM citrate buffer, pH 6.0 or 1X EDTA buffer, pH 8.0 at 120 °C for 20 minutes. Blocking was performed with 1% (wt/vol) bovine serum albumin plus 10% normal goat serum at room temperature for 60 min. After blocking, sections were incubated overnight with the selected primary antibodies at 4 °C. After washes with Tris-buffered saline containing 0.05% Tween 20, sections were incubated for 60 minutes at room temperature with Alexa Fluor 647 (1:500; Abcam, cat# ab150075) secondary antibodies. Nuclei were counterstained with 300 μM DAPI (ThermoFisher, cat# D1306) for 4 min. Slides were mounted with EMS mounting medium (Electron Microscopy Sciences, cat# 17989–50), the covers applied and held in place with clear nail polish. Secondary antibody only sections were subjected to the same procedures except blocking buffer only was added in place of the primary antibody for the overnight incubation. Imaging of slides was performed with a Nikon Widefield microscope with 20X lenses. All ovary sections were imaged using the same camera settings across WT and *GalT*KO samples for each antibody.

Background autofluorescence from secondary antibody-only controls were subtracted from primary antibody-stained samples using NIS-Elements Analysis software (Nikon Corporation, Americas). Follicle and cell types were sorted in the DAPI channel by their morphology, with the oocytes and granulosa cells analyzed separately for each follicle type. Oocytes were differentiated from granulosa cells by separate regions of interest in follicles where visible nuclei were present. Staining intensities were quantified in oocytes and granulosa cells of different classes of follicles by measuring average signal intensity in the region of interest; for ɣ-H2AX and CCASP3 in secondary follicle granulosa cells, individual stained cells were counted, and the ratio of positive stained cells compared to the total area of the ovary sample measured, instead of intensities averaged. For average intensities calculated, the signal was quantified and divided by the area for each region of interest.

### Follicle counts

One ovary from separate animals at either 14D or 30D was harvested and fixed in Dietrich’s fixative for 12 hours, rehydrated and embedded as described above. Pole-to-pole serial sections of 5 μm were cut and placed on slides by AML Laboratories (St. Augustine, FL, USA) or the Research Histology Core Facility of the ARUP Laboratories (Salt Lake City, UT, USA). Sections were then stained with Weigert’s Iron Hematoxylin (Electron Microscopy Services, cat# 26044–05) and Picric Acid (Electron Microscopy Services, cat# 26853–07)-Methyl blue (Thermo Fischer Scientific, cat#H37721–21) with cover slides mounted using Permount (Fischer Scientific, cat# SP15–100). Each sample was assigned a unique code for blinding purposes.

Blinded histomorphometric evaluation of ovarian follicles was performed [37]. The methods are described briefly as follows. Blinded to genotype, intact immature (primordial, primary, and small preantral) follicles were counted in every fifth section by the same individual, and the total numbers were estimated by multiplying by five. Adjacent sections were viewed to ensure these structures were only counted once. In addition to counting the standard follicle classes, sections were evaluated for any follicular abnormalities. After evaluation, ovaries were decoded, and data were analyzed as WT verses mutants for each age group.

### Statistical analysis

Statistical significance was determined as indicated, by using t test or Mann-Whitney U test (MW-U), and Kolmogorov-Smirnov (KS) test for non-parametric data and distributions, respectively. The statistical program R was used to analyze and graph signal intensity distributions with KS test. GraphPad Prism version 9.0 for Windows was used for all other statistical tests and graphing. A *p* value of less than 0.05 was considered statistically significant.

## Results

### Granulosa cells in primary and secondary follicles of mutant ovaries show alterations in markers of DNA damage/repair, translational control, follicular activation, and apoptosis at early progressive postnatal ages

Granulosa cells of primary and secondary follicles in ovaries isolated at 5D, 14D, and 30D (see Table 1 for the exact number of replicates from separate animals for each molecular marker, per figure) were assessed using immunofluorescence imaging to determine if molecular signaling differences exist in growing follicles. The time points (5D, 14D, and 30D) were selected to capture very early formation of primordial follicles, early preantral follicle development, and early antral follicle development just prior to sexual maturity in our mice, respectively [38]. We selected markers of the activated ISR (P-eIF2ɑ), cellular stress (endoplasmic stress response marker BiP), double-stranded DNA damage marker (ɣ-H2AX), follicle growth/granulosa cell division (Ki67), and the onset of apoptotic cell death (CCASP3). Only follicles with a visible oocyte nucleus were measured. Fluorescence intensities were compiled by genotype, and data were compared with t test or MW-U. Where appropriate, data were plotted as histogram frequency distributions and compared with the KS test to evaluate potentially dynamic protein target levels in oocytes, or granulosa cells. Table 2 provides a comprehensive summary of all results for the molecular markers measured.

**Table 2:** Comprehensive results for all molecular markers measured at the noted ages. Up and down arrows indicate significantly increased and decreased staining intensities compared to WT, respectively. Horizontal arrows indicate no difference between mutants and WT

Staining Intensities of ***GalT***KO Molecular Markers Compared to WT		
Postnatal Age	Granulosa Cells	Primordial Oocytes
**5 Days**	↑ BiP (Primary) ↔ ɣ-H2AX (Primary) ↓ p-eIF2ɑ (Primary) ↑ Ki67 (Primary)	↔ BiP ↓ ɣ-H2AX ↓ p-eIF2ɑ ↔ Ki67
**14 Days**	↔ BiP ↑ ɣ-H2AX (Secondary) ↓ p-eIF2ɑ (Primary and Secondary) ↑ Ki67 (Primary) ↑ C-Caspase 3 (Secondary)	↔ BiP ↓ ɣ-H2AX ↔ p-eIF2ɑ ↑ Ki67
**30 Days**	↓ BiP (Primary) ↔ ɣ-H2AX ↔ p-eIF2ɑ ↔ Ki67 ↔ C-Caspase 3 (Secondary)	↑ BiP ↑ ɣ-H2AX ↓ p-eIF2ɑ ↔ Ki67

#### Primary follicle granulosa cells: 5D

**Fig. 2:**
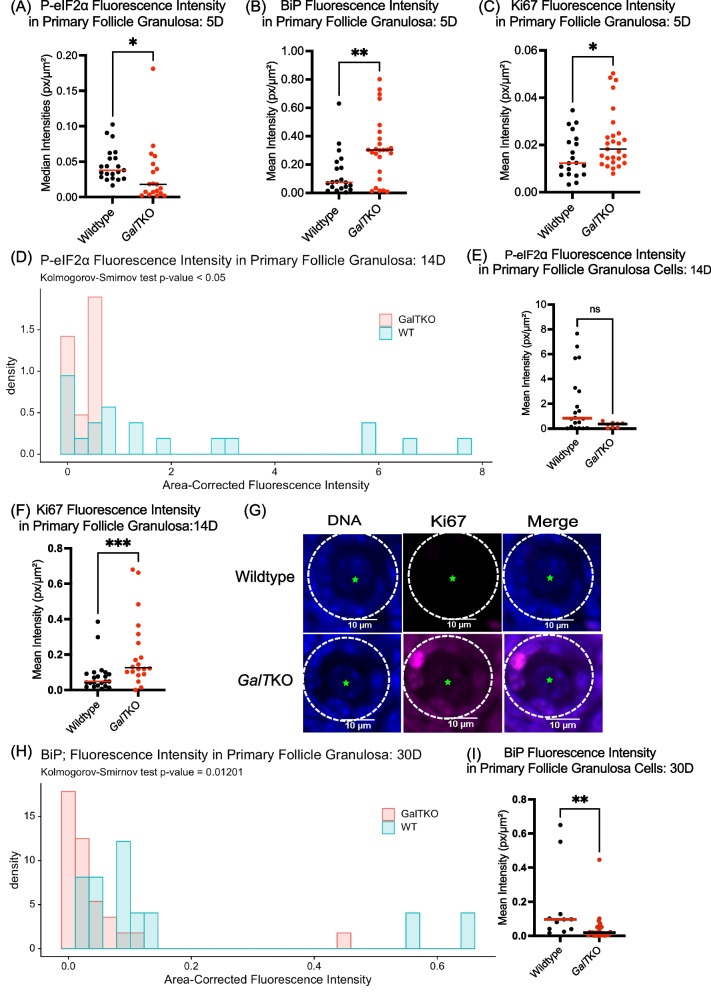
*GalT*KO primary follicle granulosa cells show increased growth and stress with impaired ISR at progressive ages. Dots plotted represent individual follicle granulosa cells measured in each specimen, compared with MW-U; horizontal lines show median values. Frequency distributions are compared with KS. Intensities for (**A**) P-eIF2ɑ (*p* = 0.01), (**B**) BiP (*p* = 0.0059), and (**C**) Ki67(*p* = 0.03) at 5D. Frequency distribution and median values of (**D**, **E**) P-eIF2ɑ (*p* < 0.05, *p* = 0.06) at 14D. At 14D, fluorescence intensities for (**F**) Ki67 (*p* = 0.0008) and representative image of a primary follicle granulosa cell staining (**G**) in WT and *GalT*KO ovaries. Circles indicate outer edge of primary follicle granulosa cells with green stars showing the oocyte. Frequency distribution of (**H**, **I**) BiP (*p* = 0.02, *p* = 0.003) at 30D.

First, we found that at 5D, *GalT*KO primary follicle granulosa cells had lower levels of P-eIF2ɑ (*p* = 0.01, MW-U, Fig. 2A), albeit elevated BiP, (*p* = 0.0059, MW-U, Fig. 2B), and increased cell proliferation marker Ki67 (*p* = 0.0331, MW-U, Fig. 2C) compared to their WT counterparts. Replicates for this and subsequent experiments are provided in Table 1.

#### Primary follicle granulosa cells: 14D

At 14D, P-eIF2ɑ was again lower in primary follicle granulosa cells in *GalT*KO ovaries (*p* < 0.05, KS, Fig. 2D,E) compared to WT. Depiction as a histogram allows appreciation that *GalTKO* primary granulosa cells consistently had very low P-eIF2ɑ levels, but WT cells were both higher and more variable. There was no difference between mutants and WT for BiP in primary follicle granulosa cells. As at 5D, average staining intensity for Ki67 in primary follicle granulosa cells was higher at 14D in *GalT*KO ovaries (*p* = 0.0008, MW-U, Fig. 2F) than WT controls. The image in Fig. 2G shows representative staining for Ki67 in primary follicle granulosa cells from a WT and *GalT*KO ovary.

#### Primary follicle granulosa cells: 30D

At 30D, BiP was lower in *GalT*KO primary follicle granulosa cells (*p* = 0.02, KS, Fig. 2H, I) than WT. There were no differences in other markers assessed at 30D in primary follicle granulosa cells.

#### Secondary follicle granulosa cells: 14D

**Fig. 3:**
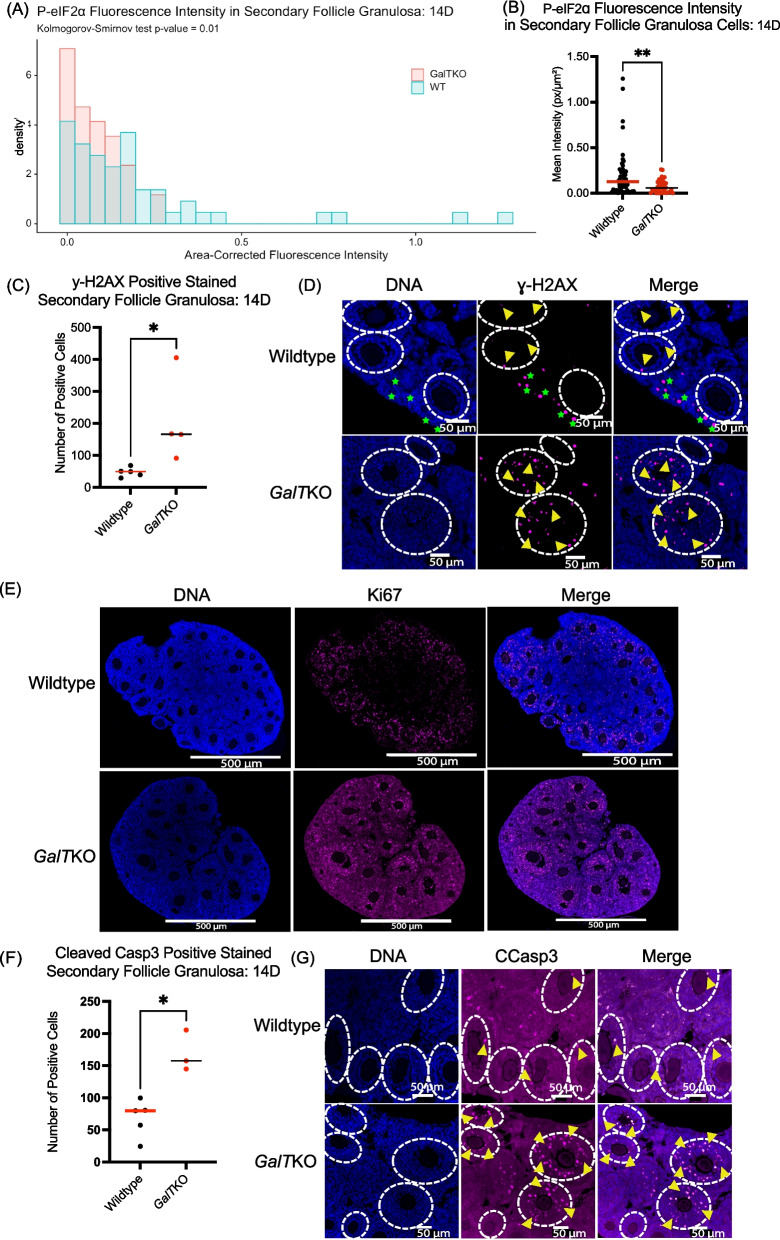
Secondary follicle granulosa cells have evidence of DNA damage/repair, increased growth, and atresia at 14D. Graph statistics include t tests, and distributions with KS test. The distribution and median of staining for (**A**, **B**) P-eIF2ɑ in secondary follicle granulosa cells (*p* = 0.01, *p* = 0.0036). **C**, **D** The number of secondary follicle granulosa cells positively stained per specimen for ɣ-H2AX (*p* = 0.03) at 14D. Yellow arrows denote positively stained granulosa cells, green stars show primordial oocytes, which only stained positive in WT samples (**D**). Global staining for (**E**) Ki67 at 14D in *GalT*KO ovaries compared to WT. Secondary follicle granulosa cells positively stained for (**F**,**G**) CCASP3 (*p* = 0.01). White dashed circles show outline of follicles

Levels of the above markers were then evaluated in the granulosa cells of secondary follicles, and here, quantification of numbers of cells positive for damage and cell death markers ɣ-H2AX and CCASP3 was performed to account for potential differences in atresia onset. As seen in primary follicle granulosa cells at 5D, the distribution of P-eIF2ɑ staining intensities was lower in *GalT*KO secondary follicle granulosa cells compared to WT (*p* = 0.01, KS, Fig. 3A, B) at 14D. The average intensity for BiP did not differ at this age in secondary follicle granulosa cells. The number of secondary follicle granulosa cells positive for ɣ-H2AX were significantly more abundant in number and per area of the ovary measured in *GalT*KO ovaries (*p* = 0.0331, t test, Fig. 3C&D) compared to WT. Green stars in Fig. 3D denote primordial follicles in WT samples, which had high staining intensities compared to very low staining in mutants at 14D. Ki67 staining intensity in secondary follicle granulosa cells did not significantly differ at 14D. Notably, “global” staining for Ki67 was higher in *GalT*KO ovaries in all ovarian cell types, while WT staining for Ki67 was nearly exclusive to granulosa cells in mainly secondary follicles (images Fig. 3E); this was consistent across all specimens. Additionally, at 14D, *GalT*KO ovaries had significantly more secondary follicle granulosa cells positive for CCASP3, per total area of ovary measured (*p* = 0.0113, Welch’s t test, Fig. 3F&G) than WT.

#### Secondary follicle granulosa cells: 30D

Finally, we extended our study to 30D to account for the mid-juvenile period, but no significant differences were detected between *GalT*KO and WT ovaries in staining intensities (P-eIF2ɑ, Ki67) or the number of positive cells (ɣ-H2AX, CCASP3) in secondary follicle granulosa cells.

### Differences are present in ɣ-H2AX, BiP, P-eIF2ɑ, and Ki67 staining in *GalT*KO primordial oocytes early in postnatal life

Primordial follicle oocytes were identified and assessed separately from granulosa cells at the noted ages. For primordial oocytes, staining intensity was the measurement for all target markers.

#### Primordial oocytes: 5D

**Fig. 4:**
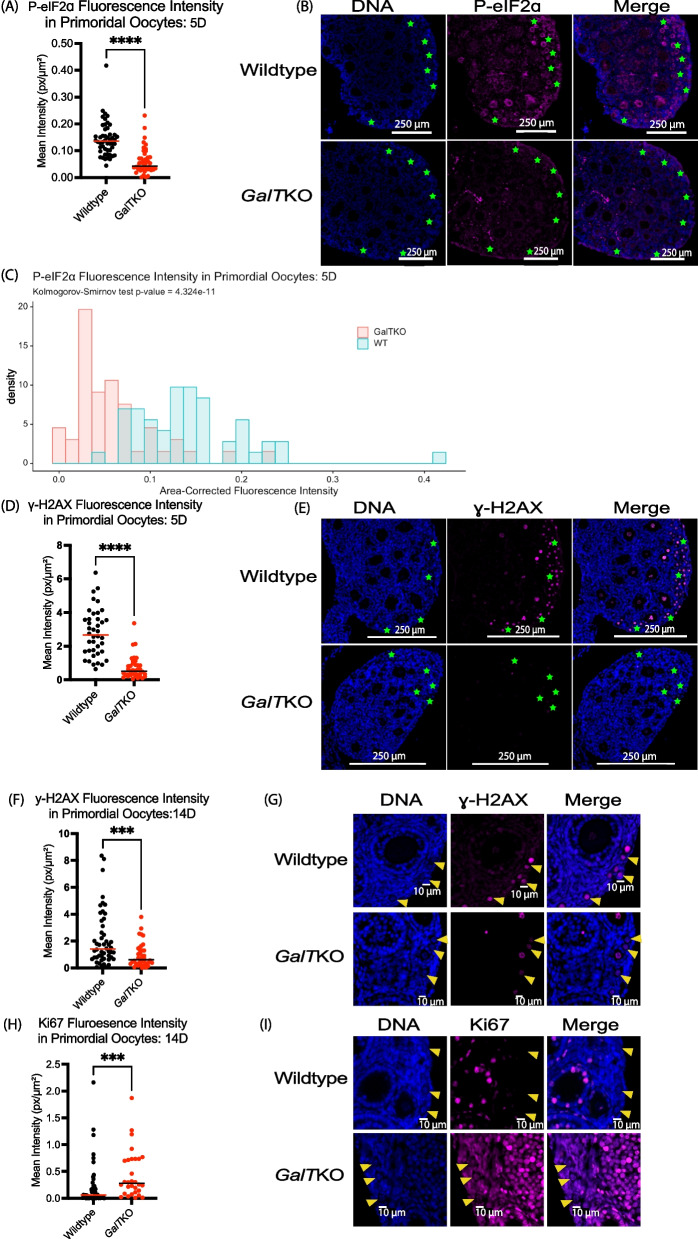
*GalT*KO primordial oocytes have marked decreases in DNA damage/repair and ISR signaling at 5D and 14D. Dots plotted represent individual primordial oocytes measured; statistics for graphs are MW-U with horizontal lines showing median values. KS test for frequency distributions. Primordial oocytes (**A**-**C**) for P-eIF2ɑ at 5D are depicted as a plotted graph, image, and frequency distribution (both graphs *p* = < 0.0001). **D**, **E** Show staining for ɣ-H2AX at 5D (*p* < 0.0001), green stars indicate clusters of primordial oocytes. **F**, **G** Show ɣ-H2AX staining at 14D (*p* = 0.0004); yellow arrows depict primordial oocytes. Staining intensity (**H**, **I**) for Ki67 in primordial oocytes at 14D (*p* = 0.0002)

*GalT*KO primordial oocytes from 5D mouse ovaries revealed dramatically lower staining intensities of ISR effector P-eIF2ɑ (*p* < 0.001, MW-U, Fig. 4A&B) compared to their WT counterparts. For P-eIF2ɑ, the distributions of individual oocyte signals were compared to capture the differences across the ovary of the primordial oocytes measured again at 5D and were highly significantly different (*p* = < 0.0001, KS, Fig. 4C). Staining for DNA damage/repair marker ɣ-H2AX (*p* < 0.0001, MW-U, Fig. 4D&E) was also much lower in *GalT*KO primordial oocytes at 5D compared to WT.

#### Primordial oocytes: 14D

At 14D, fluorescence intensity for ɣ-H2AX was again lower in *GalT*KO mouse primordial oocytes compared to WT (*p* = 0.0004, MW-U, Fig. 4F&G). Ki67 was also significantly elevated in *GalT*KO mouse primordial oocytes at this age (*p* = 0.0002, MW-U, Fig. 4H&I).

#### Primordial oocytes: 30D

**Fig. 5:**
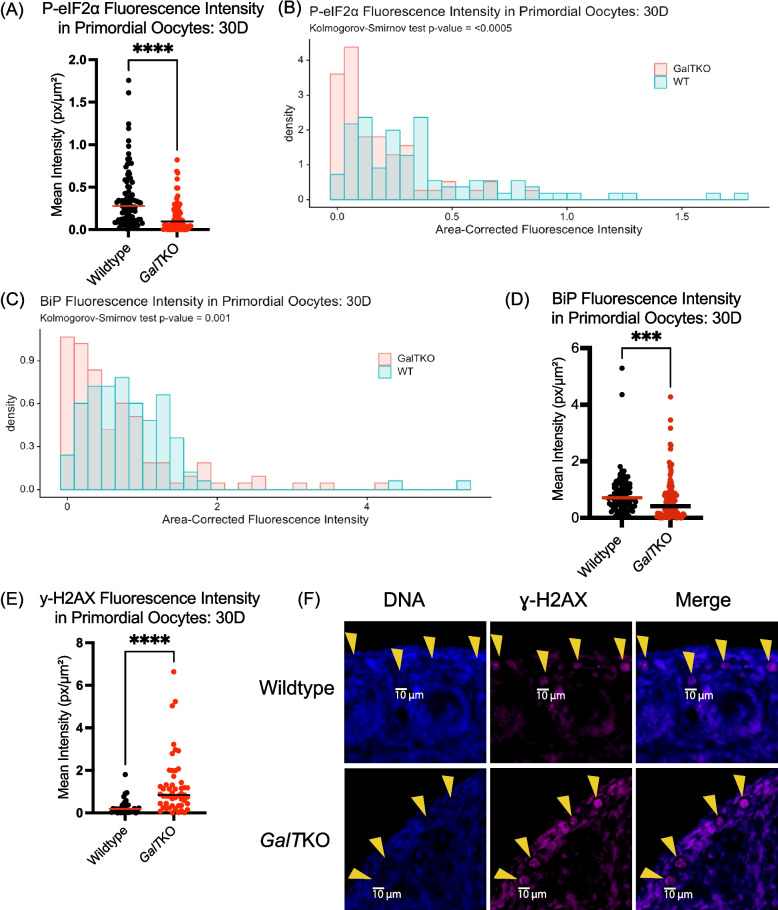
At 30D, *GalT*KO primordial oocytes had increased DNA damage, and decreased translation control. Staining (**A**,**B**) for P-eIF2ɑ at 30D (*p* < 0.0001, *p* = 0.0005). Intensities of individual primordial oocytes (**C**, **D**) for BiP at 30D (*p* = 0.001, *p* = 0.0007). **E**, **F** Shows ɣ-H2AX staining in *GalT*KO primor- dial oocytes at 30D (*p* = 0.0002). Yellow arrows show individual primordial oocytes (**F**)

Fluorescence intensity for P-eIF2ɑ remained lower in *GalT*KO primordial oocytes at 30D (*p* < 0.0001, MW-U, Fig. 5A, KS, Fig. 5B). Staining intensity for BiP was also significantly lower in *GalT*KO primordial oocytes compared to WT (*p* = 0.001, KS, Fig. 5C, MW-U, Fig. 5D) at 30D. In contrast to 5D and 14D primordial oocytes, *GalT*KO primordial oocytes at 30D instead had increased staining intensity for ɣ-H2AX (*p* = 0.0002, MW-U, Fig. 5E&F) levels compared to WT.

### Histomorphometric analyses reveal altered follicle recruitment and compromised secondary follicle survival in the juvenile *GalT*KO ovary

**Fig. 6:**
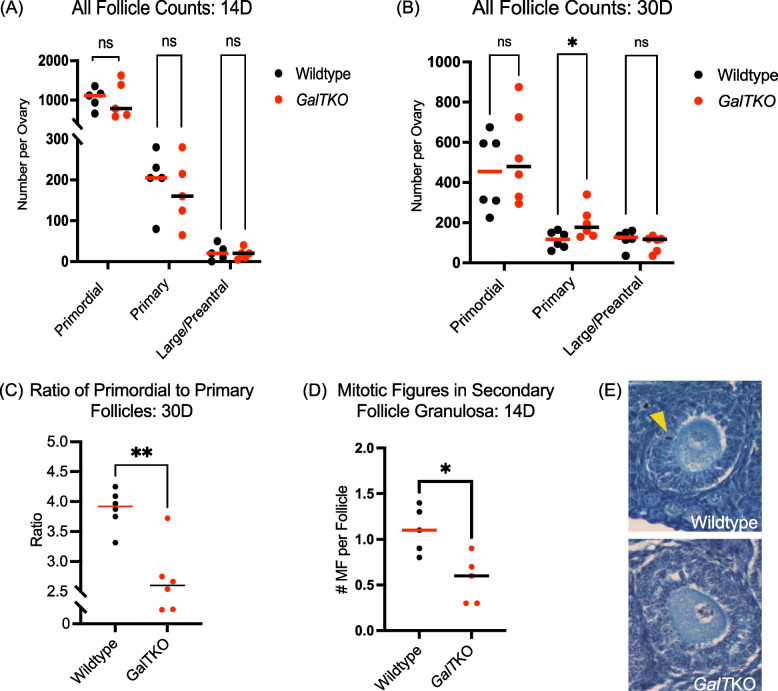
Follicle counts at 30D show a greater number of primary follicles and less evidence of growth in secondary follicles in *GalT*KO ovaries. **A**, **B** All follicle counts at 14D and 30D (primary follicles at 30D, *p* = 0.04). At 30D, (**C**) the ratio of primordial to primary follicles (*p* = 0.001). **D** Mitotic figure counts per ten follicles, per ovary (*p* = 0.01). The yellow arrow highlights an example of a mitotic figure in a WT specimen compared to no present mitotic figures in a *GalT*KO (**E**)

Primordial, primary, and large/antral follicles were counted in serial sections from 14D and 30D ovaries to capture the rate of follicle loss in a temporally overlapping fashion with the assessed stress and damage markers above. Counts at 5D were not quantified as previously counted as follicles at 8D showed no differences between mutants and WT (unpublished data). In addition, mitotic figures in ten small secondary and larger preantral follicles per ovary, per genotype, were quantified at 14D to assess the proportion of growing follicles, as we found increased evidence of growth and subsequent death in the molecular markers in *GalT*KO secondary follicles at 14D. While no differences were present in primordial, primary, and large/antral follicles at 14D (Fig. 6A) and no differences in number of primordial and large/antral in 30D ovaries (Fig. 6B), *GalT*KO ovaries had significantly more primary follicles (*p* = 0.04, t test, Fig. 6B) and a significantly lower ratio of primordial to primary follicles at 30D compared to WT ovaries (*p* = 0.001, unpaired t test, Fig. 6C). The number of mitotic figures per secondary and preantral follicles at 14D was lower in mutant ovaries compared to WT (*p* = 0.01, t test, Fig. 6D).

## Discussion

POI is a devastating complication in human CG patients, with most females affected and no preventative treatment. The molecular mechanisms contributing to follicle loss and ovarian insufficiency in CG specifically are only now being elucidated. In our *GalT*KO mouse model, there are marked decreases in primordial follicles at 6 months of life, longer times to pregnancy, and fewer litters and number of pups born per litter [25], making this a physiologically relevant model to study the metabolic basis of POI. We previously showed the involvement of PI3K/AKT signaling, endoplasmic reticulum stress, and aberrant ISR in our *GalT*KO mouse model [27, 30]. Here we expand our knowledge of involved signaling pathways and the timing of molecular changes in the *GalT*KO mice.

Activation of the ISR by cellular stress and damage (including DNA damage) leads to a cascade of cellular signaling that promotes a state of cytoprotection and cell cycle arrest until the damage can be resolved. This arises due to the ISR signaling convergence upon eIF2ɑ phosphorylation, which slows global protein translation, but favors the translation of reparative proteins. In the normal ovary, it appears that baseline activation of the ISR is integral in follicle dormancy and arrest [30]. For example, ER stress upstream of the broader ISR occurs under normal physiologic conditions in both dormant primordial and growing follicles [39]. While cellular stress and damage can be considered natural aspects of folliculogenesis, alterations in the levels of stressors occur in disease states that impact the ovary, as seen in polycystic ovarian syndrome and in obesity [40, 41]. In this study, we detected variations in stress and damage markers in cells of young *GalT*KO ovarian follicles compared to WT controls. This is summarized as follows: Immature follicles within *GalT*KO ovaries exhibit altered ISR activity (Figs. 2A&D, 3A, 4A-C, 5A&B), DNA damage/repair (Figs. 3B&C, 4D-G, 5A&B), small follicle growth (Figs. 2C, E&F, 3D, 4H&I, 6A-D) and cellular stress/death (Figs. 2B&G, 3E&F, 5C).

Specifically, we saw evidence of increased ER stress response marker BiP in primary follicle granulosa cells at 5D and then decreased BiP in primary follicle granulosa cells and primordial oocytes at 30D in *GalT*KO ovaries. Consistent with elevated levels of BiP in primary follicle granulosa cells at 5D, a *GALT-*knockout granulosa cell line *(GALT*KO OV3121) developed by Rushing and colleagues also exhibits increased BiP at baseline in those cells compared to the parent cell line (personal communication). Decreased BiP at 30D in primordial oocytes and primary follicle granulosa cells may indicate mutant ovaries at this age have a dysfunctional stress and growth response [20], but further analysis is needed.

The concomitant increase of growth marker Ki67 at 5D and 14D in primary follicle granulosa cells reveals what is likely to be accelerated follicle growth in *GalT*KO ovaries; this is matched by significantly more primary follicles and a significantly lower ratio of primordial to primary follicles at 30D in *GalT*KO ovaries. Lower staining intensity for P-eIF2ɑ in primary and secondary follicle granulosa cells at 5D and 14D (Figs. 2A, 3A) is also consistent with accelerated follicle growth at the level of granulosa cell proliferation, as again, phosphorylated eIF2ɑ favors cell cycle arrest [31]. The mechanisms that account for the *lower* P-eIF2ɑ despite elevated stress markers like BiP (above) and elevated DNA damage (below) in *GalT*KO ovarian follicles remains unclear.

Cells of *GalTK*O ovarian follicles also show an increase in the DNA damage/repair marker ɣ-H2AX and the apoptosis onset marker CCASP3. This was found in the granulosa cells of secondary follicles at 14D at the same time as the detected decrease in P-eIF2ɑ. Increased staining for these markers, along with decreased mitotic figures as follicles reach the secondary stage, are consistent with observations in humans with CG that secondary and preantral follicles appear fewer and arrested as early as childhood [23]. Secondary follicle granulosa cells may not be able to elicit an effective repair response in galactosemic conditions, and thus are more likely to succumb to atresia at this stage. Studies in rats treated postnatally to achieve hypergalactosemic conditions support this as the administration of galactose seems to have the greatest effect on growing follicles, with no differences in primordial or primary number, but fewer preantral follicles [42].

In the primordial oocytes, we detected significant differences as early as 5D in *GalT*KO ovaries. Decreased staining for P-eIF2ɑ in mutant primordial oocytes suggests GALT-deficiency leads to loss of constitutive ISR activation in the primordial oocyte. We also saw significantly decreased DNA damage and repair marker ɣ-H2AX in mutant primordial oocytes at 5D and 14D days but increased at 30D. This is an unexpected finding, as ɣ-H2AX foci are primarily considered markers of stress/DNA damage and subsequent cell death. However, it could indicate inappropriate DNA damage repair mechanisms, or perhaps resolution of prophase 1 of meiosis [43–46]. Marangos and Carroll found that oocytes in prophase 1 were able to continue growing to metaphase 1 even in the presence of low levels of DNA damage, which may be related to poor DNA repair response by ataxia telangiectasia mutated (ATM), which is a key player in the ISR to initiate DNA damage repair, cell checkpoint upregulation, and phosphorylate ɣ-H2AX in developing oocytes [30, 47]. To determine the mechanistic consequences of lower ɣ-H2AX in young *GalT*KO primordial oocytes, we will need to perform additional experiments by measuring other downstream targets of the ISR and related DNA repair pathways. At 30D, *GalT*KO primordial oocytes had increased ɣ-H2AX, as might be seen in oocytes exposed to DNA-damaging stimuli [48]. This difference from 5D and 14D days could be explained by the increased time and accumulation of toxic metabolites in older *GalT*KO ovaries.

## Conclusions

**Fig. 7:**
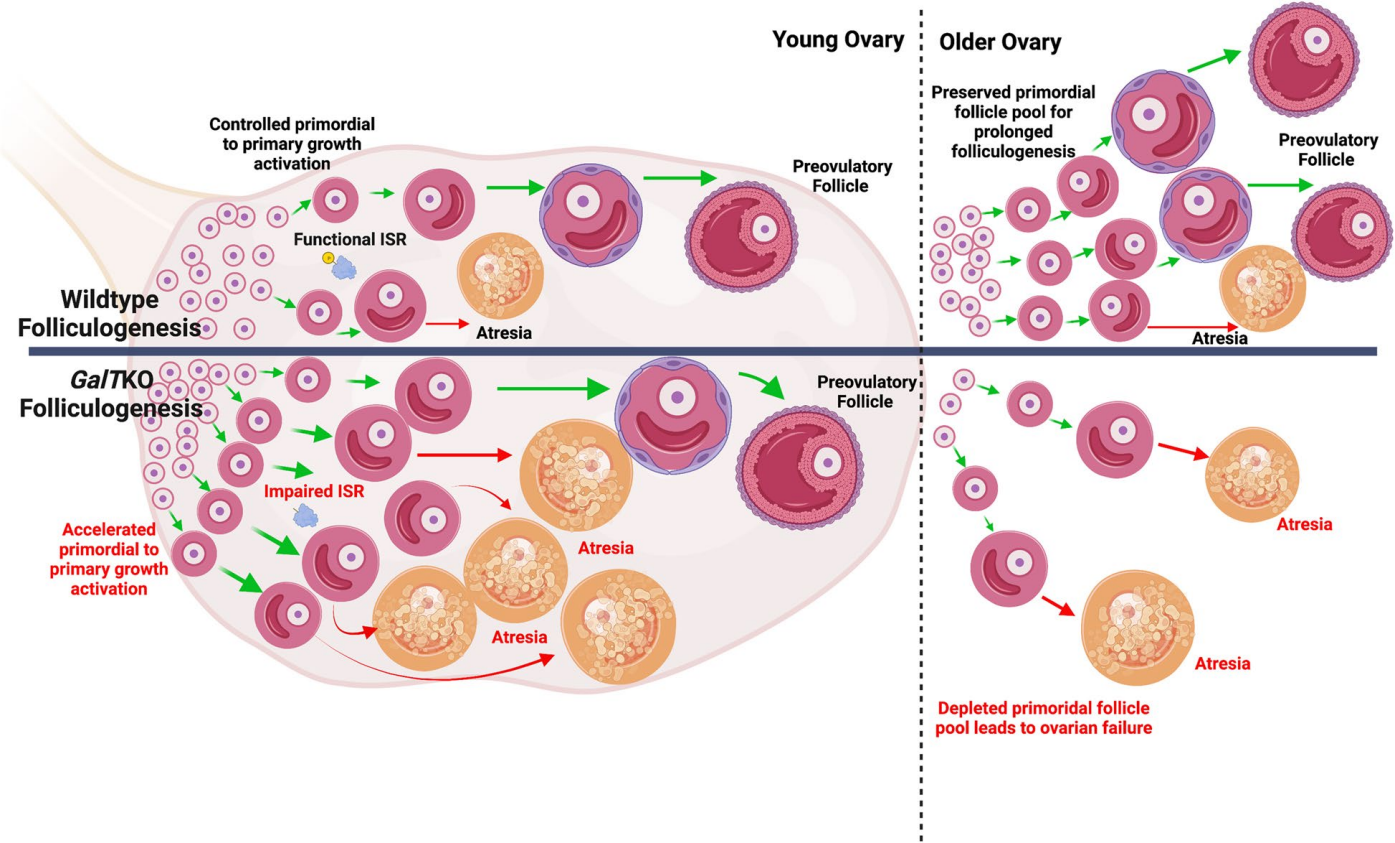
An illustrated model of follicle loss (or burnout) in *GalT*KO mice compared to WT. In *GalT*KO ovaries, early mass activation of primordial follicles leads to increased primary follicles and more atretic secondary follicles. Some follicles do achieve preovulatory status; however, as the ovary ages, fewer primordial follicles are available for growth activation. Thus, ovarian aging is accelerated and results in POI

Galactosemic conditions result in quite dynamic alterations in follicular damage and repair markers in immature ovarian follicles early in life and these plausibly contribute to the *GalT*KO POI phenotype. Based on the data above, we propose the following model: Impaired ISR action due to GALT deficiency results in an accelerated transition of primordial to primary follicles. Increased follicle growth is matched by increased atresia in secondary follicles at young ages in *GalT*KO ovaries. This may be occurring in a way that depends upon levels of potentially toxic metabolites that accumulate, and this is a key focus of ongoing studies. Accelerated loss of the primordial follicle pool is a situation reminiscent of follicle “burnout” [24] that has been reported to occur in response to toxic (e.g., chemotherapeutic) insults (Fig. 7). The absence of elevated phosphorylated ISR core regulator eIF2α, despite the evidence of increased of ER stress and DNA damage markers at all time points evaluated in this study, further supports the concept that an inability to mount a normal cytoprotective ISR response is an important feature of galactosemic ovarian dysfunction. Our prior studies that used Salubrinal to block eIF2α dephosphorylation were likely effective due to ISR function “rescue” in the mouse model [7], which protected the ovarian reserve and normalized fertility and fecundity. We now hypothesize that similar dysregulation may occur within ovarian follicles early in life in human patients with CG and that similar events contribute to early onset POI in affected adolescents and women.

## Abbreviations

CG: Classic Galactosemia
POI: Primary ovarian insufficiency
ISR: Integrated Stress Response
Gal-1P: Galactose-1 phosphate
AMH: Anti-Mullerian Hormone
FSH: Follicle Stimulating Hormone
HRT: Hormone Replacement Therapy
*GalT*KO: *GalT* gene-trapped mouse, mutant mouse
WT: Wildtype mouse
(P)-eIF2ɑ: (Phospho) eukaryotic transcription initiation factor alpha at Serine 51
CCASP3: Cleaved Caspase-3
ɣ-H2AX: Phospho-histone H2A.X
Ki67: proliferation marker protein Ki67
MW-U: Mann-Whitney U test
KS: Kolmogorov-Smirnov statistical test
